# Hiccups, Hypersalivation, Hallucinations in Parkinson’s Disease: New Insights, Mechanisms, Pathophysiology, and Management

**DOI:** 10.3390/jpm13050711

**Published:** 2023-04-23

**Authors:** Vinod Metta, Guy Chung-Faye, Hani TS Benamer, Rukmini Mrudula, Vinay Goyal, Cristian Falup-Pecurariu, Neha Muralidharan, Desh Deepak, Mohammed Abdulraheem, Rupam Borgohain, Kallol Ray Chaudhuri

**Affiliations:** 1 Department of Neurosciences, Institute of Psychiatry, Psychology & Neuroscience and Parkinson’s Foundation Centre of Excellence, King’s College Hospital, King’s College London, London WC2R 2LS, UK; 2 Kings College Hospital London, Dubai 263267, United Arab Emirates; 3 Department of Movement Disorders & Parkinson’s Centre of Excellence, Mohammed Bin Rashid University, Dubai 263267, United Arab Emirates; 4 CNC Institute of Movement Disorders & Parkinson’s Centre of Excellence, India; 5 Institute of Movement Disorders, Medanta Hospitals, India; 6 Department of Neurology, Transilvania University Brasov, 500001 Brașov, Romania

**Keywords:** Parkinson’s disease, hiccups, hypersalivation, hallucinations, botulinum toxin

## Abstract

Parkinson’s disease (PD) is a chronic, progressive neurological disorder and the second most common neurodegenerative condition. We report three common but overlooked symptoms in PD—hiccups, hypersalivation, and hallucinations—in terms of their prevalence, pathophysiology, and up-to-date evidence-based treatment strategies. Whilst all these three symptoms do occur in many other neurological and non-neurological conditions, early recognition and treatment are paramount. Whilst hiccups affect 3% of healthy people, their rate of occurrence is higher (20%) in patients with PD. Hypersalivation (Sialorrhea) is another common neurological manifestation of many neurological and other neurodegenerative conditions such as motor neuron disease (MND), with a median prevalence rate of 56% (range: 32–74%). A 42% prevalence of sialorrhea is also reported in sub-optimally treated patients with PD. Hallucinations, especially visual hallucinations, are commonly reported, with a prevalence of 32–63% in PD, and a 55–78% prevalence is noted in patients with dementia with Lewy bodies (DLB), followed by tactile hallucinations, which are indicated by a sensation of crawling bugs or imaginary creatures across the skin surface. Whilst mainstay and primary management strategies for all these three symptoms are carried out through history taking, it is also essential to identify and treat possible potential triggers such as infection, minimise or avoid causative (such as drug-induced) factors, and especially carry out patient education before considering more definitive treatment strategies, such as botulinum toxin therapies for hypersalivation, to improve the quality of life of patients. This original review paper aims to provide a comprehensive overview of the disease mechanisms, pathophysiology, and management of hiccups, hypersalivation, and hallucinations in Parkinson’s disease.

## 1. Hiccups in Parkinson’s Disease (PD)

### 1.1. Definition: What Are Hiccups?

Hiccups are the uncontrollable contractions of the diaphragmatic muscle, triggered after a spontaneous closure of the vocal cords or glottis, producing a ‘hic’ sound [1]. They are classified as persistent or intractable if they last for >2 days or >30 days, respectively.

### 1.2. Prevalence of Hiccups

#### Prevalence in General vs. in Parkinson’s Disease—Methodology

Although hiccups affect 3% of healthy people, their rate of occurrence is higher (20%) in patients with Parkinson’s disease and those on dopamine agonists [2,3]. The evidence further reveals a prevalence of 0.5% in patients with cancers undergoing chemotherapy (specifically with platinum-based compounds such as cisplatin and carboplatin, etc.). Findings from recent studies have indicated a 6.1–10% incidence of hiccups following the administration of cisplatin to Japanese patients [3]. Another prospective study revealed a 41.2% incidence of hiccups in patients with cancer who underwent cisplatin treatment [4]. However, no evidence to date has comparatively analysed the incidence or prevalence of hiccups among patients with PD, cancers, and other chronic diseases. We performed a systematic literature search using standard electronic databases of published surveys, all published case–control studies, all recent published reviews, and original research papers.

### 1.3. Pathophysiology of Hiccups

Whilst in the general population, hiccups may be associated with gastrointestinal diseases such as reflux esophagitis, they also sometimes develop in patients with a large hiatus hernia [5]. While conditions such as foreign bodies in the laryngeal, pharyngeal, and nasal passages activate hiccups, cardiovascular causes include aortic aneurysm, coronary artery disease, pericarditis, and myocardial ischemia [6]. Alternatively, dopamine agonists, especially D3 agonists, which cause alterations in the serotonergic pathway or prolonged stimulation of dopamine-3 receptors, are believed to activate hiccups in Parkinson’s disease [7].

**Figure 1:**
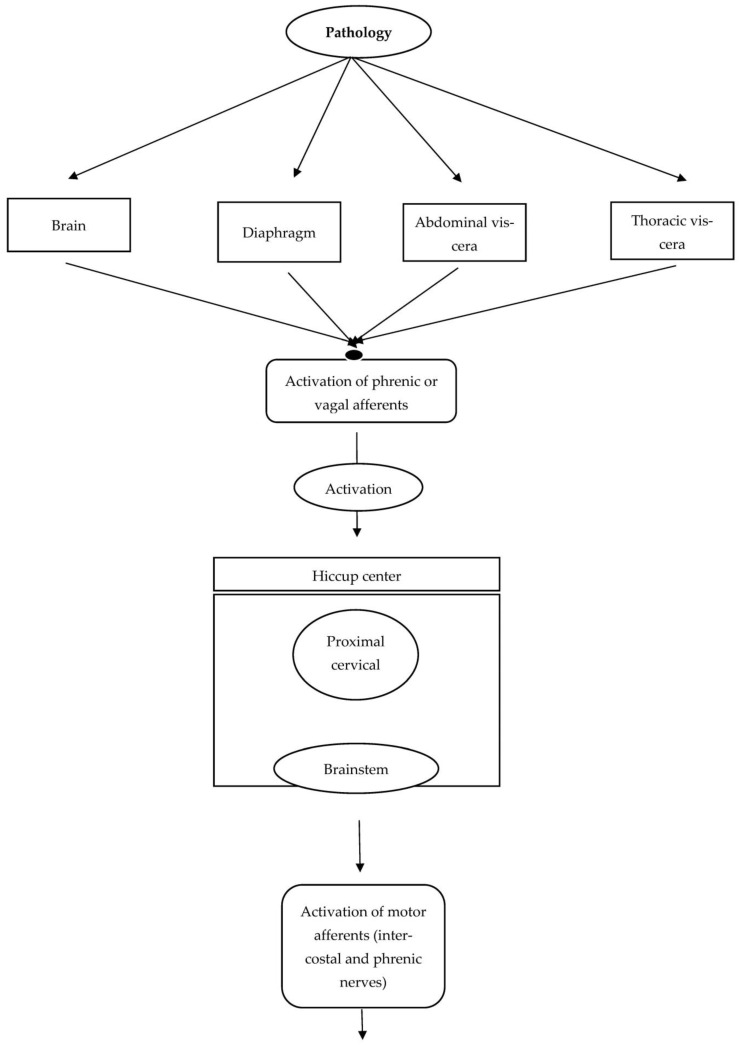

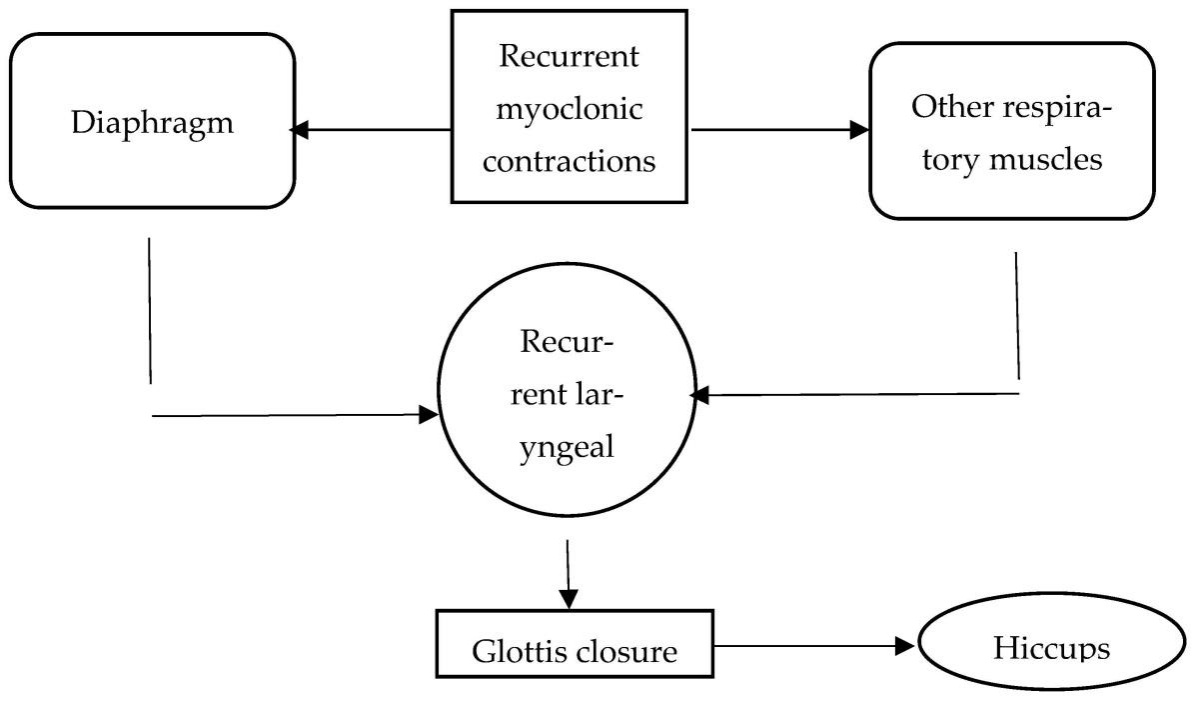
Anatomy and pathology of the hiccups reflex arc.

In addition, hiccups may also develop due to brainstem lesions or neurodegenerative conditions such as α-synucleinopathies. Anti-Parkinson’s drugs or brainstem lesions are supposed to impact one or more of the three components of the hiccup reflex arc. These components include the efferent nerve (traversing intercostal muscles, accessory nerves, diaphragm, and phrenic nerves), the midbrain’s central processing unit, and the afferent limb, which relays visceral, sensory, and somatic signals via sympathetic, vagus, and phrenic nerves. The central mechanism of neurotransmission is controlled and modulated via GABAergic and dopaminergic neurotransmitters. Moreover, the phrenic nerve potentiates the hiccups reflex by processing the efferent response that eventually leads to bilateral or unilateral contraction of the diaphragm. Figure 1 depicts the anatomy and pathology of the hiccups reflex arc.

### 1.4. Management of Hiccups in Parkinson’s Disease

The management of hiccups involves non-pharmacological (conservative) and pharmacological management.

#### 1.4.1. Conservative Management

The conservative management of hiccups aims to eliminate their underlying cause and triggers [5]. Conservative measures include Valsalva manoeuvres, rebreathing, and the holding of breath during expiration and inspiration [8]. Cold water intake is another conservative approach to managing self-limited hiccups. Similarly, vagal simulation is performed via induced vomiting/fright, carotid massage, and face (cold) compression [9].

#### 1.4.2. Pharmacological Management

##### Hiccups in General and Cancer Patients

Gabapentin, with a dose range of 900–1200 mg per day, is recommended to treat cancer-related hiccups [10]. Evidence indicates 85% complete resolution, 15% partial resolution, and 18% treatment failure [5]. Similarly, nifedipine in the dose range of 20–60 mg per day leads to 57% complete resolution, 14% partial resolution, and 29% treatment failure in patients with idiopathic hiccups due to morbidity or surgery [5]. Other potential drugs based on anecdotal practice for hiccup management include valproic acid, orphenadrine, nimodipine, midazolam, ketamine, glucagon, carvedilol, benzonatate, atropine, olanzapine, risperidone, haloperidol, amitriptyline, and amantadine. In addition, the management of intraoperative hiccups relies on lidocaine, ketamine, dexmedetomidine, ephedrine, and atropine.

##### What Do the Guidelines Say?

NICE (National Institute for Health and Care Excellence) guidelines

NICE guidelines for hiccup management in Parkinson’s disease advocate the elimination of its primary or secondary causes and the exclusion of triggers [11]. They also advocate multidisciplinary therapies, including psychotherapy, acupuncture, and hypnotherapy, to reduce hiccup frequency in Parkinson’s disease. Other potential NICE-recommended techniques for hiccup management include phrenic nerve inhibition, pharmacotherapy, and deactivation of the hiccup reflex arc.

#### 1.4.3. Pharmacotherapy

NICE guidelines advocate avoiding drugs including metoclopramide, haloperidol, and chlorpromazine, which are contraindicated in Parkinson’s disease. The first-line treatments recommended for hiccup management in Parkinson’s disease include prokinetics such as oral domperidone (20–30 mg), along with baclofen and gabapentin [12]. Baclofen, by inhibiting gamma-aminobutyric acid (GABA) within the dose range of 5–20 mg, effectively treats hiccups in patients with Parkinson’s disease [13]. However, it should be cautiously used in patients with hepatic or renal insufficiency [2]. Gabapentin in low doses effectively modifies the transmembrane potential, which eventually leads to presynaptic inhibition in the spinal cord and brain. It can be used in cancer-related hiccups with a dose range of 900–1200 mg per day [10].

Similarly, nifedipine in the dose range of 20–60 mg per day leads to 57% complete resolution, 14% partial resolution, and 29% treatment failure in patients with idiopathic hiccups due to morbidity or surgery [5]. Other potential drugs based on anecdotal evidence for hiccup management in Parkinson’s disease include dexamethasone and amantadine. Amantadine with NMDA (N-methyl-D-aspartate) antagonists acts on the subtypes of glutamate receptors and blocks the glutamatergic transmission, which helps in controlling hiccups in Parkinson’s disease.

### 1.5. Management of Intractable Hiccups

Intractable hiccups not responding to the above measures should be managed in a high-dependency unit (HDU) or intensive care unit (ICU) using sedatives such as midazolam, ketamine, and dexmedetomidine. Table 1 depicts the medications considered safe or unsafe for Parkinson’s disease.

## 2. Hypersalivation in Parkinson’s Disease

### 2.1. Definition

Sialorrhea or hypersalivation, which is commonly referred to as drooling, is defined as the excessive pooling of saliva extending toward the margin of the lips [14].

### 2.2. Prevalence

Sialorrhea is a common neurological manifestation of many neurological and neurodegenerative conditions such as motor neuron disease, cerebral palsy, and Parkinson’s disease. The literature reveals a 21–32% prevalence of moderate to severe hypersalivation in 50–70% of patients with motor neuron disease [15]. Hypersalivation has a median prevalence rate of 56% (range: 32–74%) in the advanced or early stages of Parkinson’s disease [16]. A 42% prevalence of sialorrhea is reported in sub-optimally treated patients with Parkinson’s disease [16].

Findings from recent studies have indicated more than 50% overall prevalence of hypersalivation in patients with Parkinson’s disease. However, hypersalivation has a 6–15% prevalence in normal or diseased individuals without Parkinson’s disease. The hypersecretion of saliva in patients with Parkinson’s disease occurs through minor/sublingual (10%), submandibular (60%), and parotid (30%) glands. Parotid glands are responsible for >50% production of ptyalin-rich saliva [17].

Recent studies have indicated a 90% and 42% prevalence of hypersalivation in patients with autonomic dysfunction and untreated/very early Parkinson’s disease [17]. Some studies have indicated significant problems in 37% of patients with hypersalivation due to Parkinson’s disease and 50% prevalence in patients with Parkinson’s disease at an early stage [17]. Other studies have revealed a 43% prevalence of night-time drooling or excessive salivation sensation in 71% of patients with sialorrhea in Parkinson’s disease [17]. While 55.3% drooling prevalence has been reported at 4-year follow-up in Parkinson’s disease, 11.7% of patients developed significant hypersalivation requiring immediate management [17].

Some studies have reported a 70–80% prevalence of drooling in patients with Parkinson’s disease based on their utilization of neuroleptics, hypokinesia, or swallowing impairment. Nocturnal hypersalivation has been reported in 92% of patients who receive antipsychotic medications such as clozapine [18].

### 2.3. Pathophysiology

Normal daily production of saliva varies between 0.15–0.5 L with a flow rate of 0.3–0.4 mL/min. This rate decreases to 0.1 mL/min during sleep and increases to 0.4–0.5 mL/min during chewing and eating.

Sialorrhea or hypersalivation (when the flow of saliva exceeds 0.7 mL/min) or excessive salivation in many cases is not accompanied by an increased salivary flow. Generally, the flow of saliva is normal or reduced, and only oral distribution and handling of saliva are interrupted [19].

Normal swallowing activity relies on neuromuscular functioning and coordination of the oesophagus, larynx, pharynx, and oral cavity. There are three phases of swallowing: the first phase, oral, under voluntary control; the second, pharyngeal; and the third, oesophageal, under involuntary (autonomic) control of the swallowing centre, which is centred in the lower pons and medulla oblongata of the brainstem; more specifically, the nucleus ambiguous. Drooling from the anterior mouth is the outcome of the incoordination of the lingual musculature that inhibits the passage of saliva to the oropharynx via the mouth based on a marked reduction in the swallow reflex regulating the salivary secretion, which is controlled by the periodontal ligament (and its mechanoreceptors) and taste buds (with their chemoreceptors) [20].

In addition, the salivary nuclei of the medulla oblongata receive efferent impulses from cranial nerves that eventually control the function of sublingual and submandibular glands. The parotid gland is specifically influenced by the cranial nerve IX, which directs the secretion and elimination of saliva [20].

Sialorrhea in Parkinson’s disease is multifactorial and mainly attributable to autonomic dysfunction, while flexed head posture and improper swallowing are its predominant causes in patients with Parkinson’s disease. The lack of spontaneous drooling control in patients with Parkinson’s disease disrupts the elimination of saliva, thereby leading to its unwanted accumulation in the oral cavity [14]. Patients with Parkinson’s disease have a shorter oropharyngeal transit time and elevation in tongue pressure, which triggers drooling or hypersalivation [21]. In addition, the deterioration in the oral phase is the outcome of minimized swallowing frequency and disrupted oropharyngeal transport.

Sialorrhea rapidly progresses with disease progression, which is evidenced by worsening MDS-Unified Parkinson’s Disease Rating Scale (MDS-UPDRS) motor scores [22]. Several sialometry studies have revealed a marked reduction in the production of saliva from the salivary glands in patients with Parkinson’s disease when compared to healthy patients [23]. This in turn reduces swallowing ability, which further disrupts the swallow reflex and minimizes salivary clearance. Some studies have hypothesized the concomitant role of these disruptions in restricting the clearance of salivary secretions and oropharyngeal bradykinesia in triggering sialorrhea in Parkinson’s disease [24].

Clozapine-induced hypersalivation relates to its potential to induce alpha-2-adrenoceptor gene polymorphism [25]. It also agonizes M4/M3 glandular muscarinic and alpha-2 adrenergic receptors that eventually increase saliva production by stimulating the sympathetic and parasympathetic nervous systems.

### 2.4. Assessment

The assessment of sialorrhea includes detailed history-taking and physical and neurological examination.

### 2.5. History Taking

The assessment of sialorrhea and its severity is necessary to evaluate prognostic outcomes and quality of life [26]. Appropriate history-taking from the patient and caregiver is warranted to identify any potential triggers (such as gastroesophageal reflux and medications), the severity of the situation, and the impact on activities of daily living.

### 2.6. Physical Examination

The physical examination should investigate jaw stability, malocclusion, nasal blockage, tonsillar hypertrophy, tongue movement/size, dental problems, and chin/lip sores to identify their possible roles in hypersalivation and detailed neurological examination, including the level of alertness, bedside swallow tests, nutrition and hydration status, head posture, and the behavioural and emotional status of the patient.

### 2.7. Can We Quantify or Measure Hypersalivation?

Both subjective and objective measures have been developed and validated to quantify excessive salivation. Subjective scales include the drooling frequency and severity scale (DFSS), an easy-to-use scale that measures and rates the severity of drooling on a five-point scale and frequency on a four-point scale [27]. Other measurements rely on the Drooling ImpactScale (which is a 10-point scale to determine the frequency and severity of drooling and its impact on daily living activities) [28] and the visual analogue scale (used to measure drooling severity) [29,30]. However, the latest evidence reveals that Radboud Oral Motor Inventory for Parkinson’s Disease (ROMP) is the only valid scale to quantify the non-motor symptom (or drooling) in patients with Parkinson’s disease [31].

The objective method includes the direct observation of saliva loss by counting the number of napkins and measuring the weight of towels or dental cotton rolls [32]. The other method of objective measuring of saliva (sialometry) includes using radioisotopes (scintigraphy). Objective measurements are more sensitive in measuring saliva than subjective scales.

### 2.8. Complications

Sialorrhea causes a range of physical complications, including feeding problems that increase the risk of aspiration pneumonia, which is the major cause of mortality in patients with Parkinson’s disease [33]. The psychosocial complications include bad odour, social interaction challenges, reduced self-esteem, and social isolation [26].

### 2.9. Treatment of Sialorrhea in Parkinson’s Disease

The main goal of sialorrhea management is the reduction in salivary flow whilst maintaining oral hygiene (to avoid xerostomia). It can be challenging at times and depends on numerous factors such as the patient’s cognitive and mental status and posture of the head [14]. The two main approaches to sialorrhea treatment include the following.

#### Conservative Approach

The primary objective of conservative management is to maintain the overall health and moisture in the oral cavity while concomitantly minimizing hypersecretion [34]. The other objective includes educating patients and caregivers about swallow mechanics and posture techniques. In addition, the use of a wheelchair with the head back is often recommended to improve patient posture.

Non-invasive techniques should aim to minimize the risk and incidence of dry mouth or xerostomia. These procedures are based on botulinum toxin, pharmacological therapy, oral prosthetic devices, negative/positive reinforcement, biofeedback, speech therapy, and oral facial facilitation. Dentists and otolaryngologists should assist patients in enhancing their drinking/eating abilities along with their overall positioning for effectively treating conditions such as adenotonsillar hypertrophy, macroglossia, and other factors contributing to aerodigestive obstruction to minimize its attribution to sialorrhea.

### 2.10. Pharmacological Management

The pharmacological management of hypersalivation relies on anticholinergic drugs, including tropicamide, scopolamine, benztropine, and glycopyrrolate [26]. Orally administered glycopyrrolate (1 mg three times a day) has been proven to be a safe treatment option [14] with reduced side effects owing to its quaternary ammonium structure, which makes it difficult to cross the blood–brain barrier. Other oral options include benzhexol and benztropine. These drugs act on the autonomic (or parasympathetic) nervous system and minimize the secretion of saliva through acetylcholine downregulation. However, their use is restricted in several comorbidities, including myasthenia gravis, gastrointestinal motility disorders, obstructive uropathy, glaucoma, and cognitive problems in elderly patients [35].

In Parkinson’s disease, intraoral tropicamide films provide short-term relief from hypersalivation. Transdermal scopolamine, applied as a patch behind the ear, is well tolerated as per the outcomes of short-term studies; however, its use is limited due to side effects, including urinary retention and blurred vision [36]. Sublingual atropine drops also improve drooling and are considered safe for Parkinson’s disease [37].

### 2.11. Botulinum Therapy for Sialorrhea

The salivary glands in normal conditions are responsible for 90% of daily saliva production, approximating 1.5 L [26]. However, sublingual and submandibular glands produce approximately two thirds of salivary secretions in the basal or unstimulated state [26]. Parasympathetic stimulations potentially increase the activity of the parotid glands, which leads to a five-fold increase in the production of salivary secretions. The three prominent salivary glands induce the secretion of viscous and serous saliva. The submandibular sublingual glands produce thicker or viscous saliva, while watery or thin saliva is secreted via parotid glands following their induction from the brain. The excessive production of viscous (sticky or mucoid) saliva leads to aspiration and choking due to its incomplete clearance, while the overproduction of serous saliva leads to consistent drooling from mouth corners [38].

Botulinum toxin injections to treat sialorrhea are widely used because of their limited invasiveness and demonstrable efficacy in many patients; overall, they improve the quality of life in patients due to their low side effects [39]. The use of botulinum toxin and its efficacy in Parkinson’s disease was first demonstrated in 2000 [40].

#### How Does Botulinum Toxin Work?

Botulinum toxin is a potent neurotoxin that blocks acetylcholine and also several other neurotransmitters released from synaptic vesicles, thereby blocking cholinergic postganglionic parasympathetic fibres and reducing the production of saliva [14].

A randomized placebo-controlled trial involving over 181 patients reported that the botulinum toxin improved drooling severity in both the adult and paediatric populations [41].

The botulinum toxin is effective and minimally invasive with few side effects if given under ultrasound guidance, since submandibular glands are usually nonpalpable and the ultrasound easily identifies glandular structures for infiltration that avoids accidental damage to other anatomical structures such as facial nerves (whilst injecting the parotids) and facial vessels (whilst injecting the submandibular glands) [35].

Currently, three type A (OnabotulinumtoxinA (BOTOX^®^), AbobotulinumtoxinA (Dysport^®^), IncobotulinumtoxinA (Xeomin) and one type B (RimabotulinumtoxinB) (Neurobloc^®^/myobloc) botulinum toxins are recommended for use and have been found to be effective in treating sialorrhea [14].

### 2.12. Invasive Approaches

#### 2.12.1. Surgical Options

Surgical options are considered in patients who are unable to tolerate oral medications and the side effects of the botulinum toxins. Most commonly advocated procedures include sublingual or submandibular gland excision, submandibular duct ligation, parotid duct ligation, submandibular or parotid duct rerouting, or their various combinations, due to their higher success rates [42]. Transoral endoscopic submandibular ganglion neurectomy is a recently advocated novel procedure; however, long-term data are yet awaited [43].

#### 2.12.2. Radiation Therapy

Radiation therapy for salivary glands is preferred for elderly patients who do not qualify for surgery and those who cannot tolerate oral therapy and botulinum toxin injections. The potential complications of radiation therapy include long-lasting xerostomia and malignancies [43].

#### 2.12.3. Speech Therapy

Speech therapy aims to improve swallowing (by minimizing nasal regurgitation), enhance lip closure, and increase the tongue’s positioning, strength, and mobility [32,44]. In addition, it also increases jaw closure and stability in Parkinson’s disease, which helps improve sialorrhea and its potential complications.

#### 2.12.4. Prosthetic (Oral) Equipment

Dental appliances, chin cups, and other prosthetic equipment aim to improve swallowing, tongue position, lip closure, and mandibular stability [32]. However, the results depend on the level of patient comfort and cooperation.

#### 2.12.5. What Do the Guidelines Say?

NICE guidelines recommend the effective use of non-pharmacological techniques such as language and speech therapies to control the rate and extent of hypersalivation [45]. The recommended medications for drooling management in Parkinson’s disease include botulinum toxin A and anticholinergic drugs such as glycopyrronium bromide.

## 3. Hallucinations in Parkinson’s Disease

### 3.1. Definition and Types

Hallucinations are false sensory perceptions that appear real but do not exist and can affect any or all the five senses, namely visual, auditory, olfactory, tactile, and gustatory. Patients often see things or hear voices that do not have any existence in reality [46].

### 3.2. Prevalence (General vs. Parkinson’s Disease)

Auditory hallucinations are the most frequent perceptual symptoms in patients diagnosed with schizophrenia spectrum disorder [47]. While neurological and ophthalmological conditions contribute to the development of visual hallucinations in the elderly, their prevalence in vascular dementia and Alzheimer’s disease has been recorded as 5–14% and 11–17%, respectively [48]. Additionally, 15% to 60% of visual hallucinations are attributed to Charles Bonnet syndrome. Drug-induced hallucinations (based on cannabis dependence or alcohol use) are associated with 11% and 5–20% prevalence, respectively [49].

Visual hallucinations are highly prevalent in Parkinson’s disease and impact approximately 75% of patients with this condition [50]. Some studies have recorded a 32–63% prevalence of visual hallucinations in Parkinson’s disease, while a 55–78% prevalence has been noted in patients with dementia with Lewy bodies [48], followed by tactile hallucinations, which are indicated by a sensation of crawling bugs or imaginary creatures across the skin surface [51]. In addition, gustatory hallucination is indicated by the reporting of an abnormal taste or a bitter sensation in the oral cavity despite the absence of its relevant source in the proximity [52], whereas auditory and olfactory hallucinations have been rarely reported in Parkinson’s disease [52]. Approximately, 47% of patients with visual hallucinations experience kinetic scenes [53]. Evidence has revealed a 19% prevalence of visual hallucinations in patients with treatment modifications and 40% associated with antipsychotic usage in Parkinson’s disease [53]. Table 2 shows the prevalence of hallucinations in Parkinson’s disease based on the data from prospective studies [53].

### 3.3. Etiopathology—The Role of Neurotransmitters and Neuronal Drivers

Visual processing involves an interplay between dopaminergic, serotonergic, cholinergic, and GABAergic neurons. The disruption or interruption of the circuitry connections predisposes patients with Parkinson’s disease to visual hallucinations [57]. These neuronal network changes and thalamic drivers have been implicated in the pathogenesis of hallucinations in Parkinson’s disease. The thalamus is a key driver that potentially shifts the network control and inhibits the default mode network (DMN) [58]. The study by Zarkali et al. showed reduced white matter connectivity in posterior thalamic projections in patients with Parkinson’s hallucinations [59].

Complex visual processing associated with serotonergic receptor subtypes 5HT1B/5HT2A (densely expressed in the primary visual cortex) and 5HT3 (expressed in GABAergic neurons) modulates behavioural responses to visual inputs. These receptors modulate the release of acetylcholine, which further modulates the thalamicreticular nucleus via nicotinic and muscarinic receptors and acts as a “sensory precision signal” [60]. Overall, the disruption of serotonergic and cholinergic neurotransmission plays a key role in thalamic-driven DMN inhibition and predisposes to visual hallucinations in PD [58].

### 3.4. Management of Hallucinations in Parkinson’s Disease

#### 3.4.1. Conservative Strategies

The primary strategy for treating visual hallucinations in Parkinson’s disease is to investigate recent triggers such as infection and recent medication changes based on anticholinergics, followed by amantadine, rasagiline, dopamine agonists, monoamine oxidase (MAO) B inhibitors, entacapone, and levodopa [61].

#### 3.4.2. Psychoeducation

Psychoeducation is a valuable tool for patients and caregivers to reduce stigmatising attitudes concerning psychotic experiences. Distress related to hallucinations is crucial and causes a number of problems that need to be dealt with, and it can be alleviated by medications and psychotherapy [62].

#### 3.4.3. Cognitive Behavioural Therapy (CBT)

Studies such as that by Valmaggia et al. [63] have advocated CBT as a modestly effective treatment scheme for positive psychotic symptoms. One general limit of CBT is that it does not deal with the hallucinations as such, but deals with the actual cause of distress to the experiences. Other limitations include no effect on the negative symptoms, depression, social functioning, or reduced relapse rates. Lynch et al. [64] recommend one-to-one therapist attention as more effective compared to other therapies [65,66].

#### 3.4.4. Pharmacological Options

Cholinesterase inhibitors (e.g., rivastigmine) have shown some benefit with improved cognitive domains in patients with visual hallucinations; however, no supportive data in this context are available from randomized controlled studies [67]. Among atypical antipsychotics, the National Institute of Clinical Excellence (NICE) supports the use of quetiapine, since it is safer than other atypical antipsychotics such as olanzapine (odds ratio of mortality: 2.16 vs. 2.79) [68].

Clozapine has the strongest evidence for efficacy in treating distressing and refractory hallucinations in Parkinson’s disease. Two recent randomized controlled studies have demonstrated no worsening of Parkinson’s motor symptoms in patients treated with clozapine [64]. However, its usage is limited to specialist settings associated with a risk of agranulocytosis, and it requires weekly blood monitoring for the first 18 weeks.

Recent clinical trials have indicated the ability of novel serotonergic agents (including 5HT2A inverse agonist and pimavanserin) to minimize psychosis and visual hallucinations in Parkinson’s disease [69]. Ondansetron, a 5HT3 antagonist, is already in use as an anti-emetic based on its potential to improve persistent visual hallucinations in patients with Parkinson’s disease [70].

## 4. Conclusions

Hiccups, hypersalivation, and hallucinations are common, but are overlooked symptoms in many neurological, non-neurological, and neurodegenerative diseases such as PD. Primary management strategies that help to improve patients’ quality of life include thorough history-taking, identifying and treating impending potential triggers such as infection, minimizing or avoiding causative factors, and patient and carer education before implementing pharmacological treatment strategies such as botulinum toxin therapies for hypersalivation.

## Figures and Tables

**Table 1:** Safe and unsafe medications in Parkinson’s Disease.

Medication Type	Medication Name	Brand Name	Mechanism of Action	Safe in Parkinson’s Disease (Yes/No)
Adamantanes	Amantadine	Symmetrel	Amantadine triggers norepinephrine, which eventually enhances dopamine accumulation across the endings of the nerves in the brain regions. It also antagonizes the N-methyl-D-aspartate receptor.	Yes
Skeletal muscle relaxant	Baclofen	Lioresal Lyflex	Baclofen acts on the pre-synaptic neurons and inhibits their excitatory neurotransmitters. It further reduces spasticity by activating the postsynaptic neurons and potentiating their inhibitory neuronal signals.	Yes
Calcium channel blocker	Nifedipine	Tensipine Valni	Nifedipine acts on the cells of the vascular smooth muscles and ceases voltage-dependent L-type calcium channels.	Yes
Antiemetic	Domperidone	Motilium	Domperidone acts on the dopaminergic processes, minimizes prolactin accumulation, activates gastrointestinal peristalsis, and inhibits dopamine receptors.	Yes
Corticosteroid	Dexamethasone	NeofordexGlensoludexMartapan	Dexamethasone has a protective role in Parkinson’s disease since its administration reduces the risk of damage to the nigrostriatal dopaminergic neurons.	Yes
Benzodiazepine	Midazolam	Buccolam^®^	Benzodiazepine expands the opening of the chloride channel and increases the accumulation of GABA (γ-aminobutyric acid).	Yes
Dissociative anaesthetic	Ketamine	Ketalar	Ketamine interacts with non-N-Methyl-D-Aspartate (NMDA)/NMDA receptors and the glutamate binding regions. It also reduces the accumulation of acetylcholine by blocking the activity of muscarinic/cholinergic receptors of the cholinergic neurons.	Yes
Alpha-2 agonist	Dexmedetomidine	Precedex Hospira	Dexmedetomidine selectively blocks the presynaptic alpha-2 adrenoceptors which eventually reduces norepinephrine accumulation. In addition, it also reduces heart rate and blood pressure by minimizing sympathetic activity by inhibiting the postsynaptic alpha-2 adrenoceptors.	Yes
Prokinetic agent	Metoclopramide	Maxolon	Metoclopramide reduces the risk of levodopa-based vomiting and nausea by interrupting the central dopamine processes.	No
Antipsychotic	Haloperidol	Serenace(R)	Haloperidol actively antagonizes the corpus striatum’s postsynaptic dopamine receptors, which eventually reduces the neurotransmission of dopamine and minimizes the risk of hallucinations/delusions.	No
Atypical antipsychotic	Risperidone	Risperdal Consta	Risperidone minimizes dopamine accumulation by actively ceasing the nerve cells’ dopamine production.	No
Anticonvulsant	Valproic acid	Epilim Chrono Epilim Chronosphere Dyzantil	Valproic acid minimizes the production of histone deacetylase, ceases the voltage-gated ion channels, and increases the levels of gamma-aminobutyric acid.	No
Skeletal muscle relaxant	Orphenadrine	Norflex Norgesic	Orphenadrine ceases the activity of NMDA and histamine H1 receptors. Its muscle relaxant property assists in improving mood and reducing the incidence of skeletal muscle spasms.	No

**Table 2:** (Hallucination prevalence in Parkinson’s Disease—data from prospective studies) [53,54,55,56].

Type of Hallucination	Overall or Lifetime Prevalence (%)	Point Prevalence (%)
**Visual hallucinations**	17–50%	74% (after 20-year follow-up)
**The minor hallucinatory phenomenon in patients with de novo and untreated Parkinson’s disease**	42%	-
**Passage and presence hallucinations in patients with minor hallucinations**	57.1%	-
**Isolated passage hallucinations**	28.6%	-
**Isolated presence hallucinations**	14.3%	-
**Hallucinations with a movement feeling**	45.9%	-
**Hallucinations with imagination or a feeling of someone’s presence**	24.6%	-
**Auditory hallucinations**	4.3%	-
**Olfactory hallucinations**	11.3	-
**Perception of noxious odours, including garbage, rotten eggs, and other unpleasant odours**	18.8%	-
**Perception of fruits and flowers**	81.3%	-
**Normosmia**	4%	-
**Hyposmia**	52%	-
**Functional anosmia**	44%	-
**Gustatory hallucinations**	2.8%	-
